# Comparison of Perioperative Chemotherapy versus Postoperative Chemoradiotherapy for Operable Stomach Cancer: A Western Canadian Province Experience

**DOI:** 10.3390/curroncol28020120

**Published:** 2021-03-17

**Authors:** Adnan Zaidi, Amal Khan, Claire Duval, Kamal Haider, Osama Ahmed, Dorie-Anna Dueck, Bryan Brunet, Donald Gardiner, Shahid Ahmed

**Affiliations:** 1 Saskatoon Cancer Center, Saskatchewan Cancer Agency, University of Saskatchewan, Saskatoon, SK S7N4H4, Canada; adnan.zaidi@saskcancer.ca (A.Z.); kamal.haider@saskcancer.ca (K.H.); osama.ahmed@saskcancer.ca (O.A.); dorie-anna.dueck@saskcancer.ca (D.-A.D.); bryan.brunet@saskcancer.ca (B.B.); donald.gardiner@saskcancer.ca (D.G.); 2 College of Medicine, University of Saskatchewan, Saskatoon, SK S7N4H4, Canada; amal.khan@usask.ca (A.K.); claire.duval@usask.ca (C.D.)

**Keywords:** stomach cancer, perioperative chemotherapy, postoperative chemoradiotherapy, neutrophil to lymphocyte ratio

## Abstract

Background: The standard approaches for resectable stomach cancer are postoperative chemoradiotherapy (PCR) or perioperative chemotherapy (PC). Limited evidence is available regarding the superiority of one of the two approaches. We aimed to compare the survival of patients with operable stomach cancer who were treated with PC or PCR. Methods: In this retrospective cohort study, patients with operable stomach cancer diagnosed between 2005–2015 in the province of Saskatchewan were identified and, based on type of treatment, were placed into PCR and PC groups. A Cox proportional multivariate analysis was performed to assess independent prognostic variables, including survival advantage of PC over PCR. Results: A total of 88 eligible patients with a median age of 66 (56–71) and a male to female ratio of 1:0.44 were identified. Seventy-three (83%) patients had pathologically node positive disease. Sixty-seven (76%) patients received PCR, while 21 (24%) patients received PC. The median overall survival of the whole group was 34 months, with 38 months (95% CI 24.6–51.3) in the PCR group vs. 30 months (14.3–45.7) in the PC group (*p* = 0.29). Median relapse-free survival was 34 months (20.7–47.3) in the PCR group vs. 23 months (6.7–39.3) in the PC group (*p* = 0.20). Toxicities were comparable. On multivariate analysis, T ≥ 3 tumor (HR, 3.57 (1.39–8.56)), neutrophil to lymphocyte ratio (LNR) > 2.8 (HR, 1.85 (1.05–3.25)), and positive resection margins (HR, 1.89 (1.06–3.37)) were independently correlated with inferior survival. Conclusions: This well-designed population based cohort study suggests a lack of survival benefit of PC over PCR. Both treatment options remain viable approaches for resectable stomach cancer.

## 1. Introduction

Stomach cancer remains one of the most common forms of cancer worldwide, accounting for about 9.9% of new cancers [1,2]. It represents the second leading cause of cancer death worldwide and is the fourth most common malignancy in the world [3]. Surgery is the main curative therapy; nevertheless, substantial numbers of patients with localized cancer develop recurrence following curative surgery alone. The 5-year survival rate for surgically resected stage IIB disease is below 35% and even lower for more advanced-stage disease [1,2]. Recurrence after surgery is usually incurable and efforts have been made to improve survival by the addition of adjuvant or neoadjuvant therapy. Some of the relatively modern trials involve the use of adjuvant or perioperative therapy. The landmark U.S. Intergroup (INT-0116) study and the United Kingdom Medical Council Adjuvant Gastric Infusional Chemotherapy (MAGIC) trials demonstrated that adjuvant chemoradiotherapy or perioperative chemotherapy (PC) decreases the relapse rate and improves the survival of patients with stomach cancer, respectively [4,5].

The INT-0116 study (adjuvant chemoradiation therapy versus observation after curative gastric cancer resection) was first reported in 2001. It randomized a total of 556 patients with stage IB to IVM0 operable stomach and gastroesophageal (GE) junction cancers to postoperative adjuvant chemotherapy vs. surgery alone. The postoperative adjuvant group had a median overall survival (OS) of 36 months compared to 27 months in the surgery only group (hazard ratio (HR) for death, 1.35 [95% confidence interval (CI), 1.09 to 1.66; *p* = 0.005]) [4]. This trial established postoperative chemoradiotherapy (PCR) as a standard approach for completely resected stage IB to IVM0 gastric and GE junction cancer. This approach was widely adopted in North America.

In 2006, the MAGIC (Preoperative chemotherapy versus surgery alone for resectable gastroesophageal cancer) trial by the Medical Research Council in the UK was reported. This trial enrolled 503 patients with resectable stomach (74%), distal esophageal, or GE junction adenocarcinoma who were randomly assigned to surgery alone or surgery plus PC (three preoperative and three postoperative cycles of epirubicin, cisplatin and 5-flurouracil). When compared with the surgery group, the PC group had a higher likelihood of OS (HR, 0.75; 95% CI, 0.60 to 0.93; *p* = 0.009; 5-year survival rate, 36% vs. 23%) and of progression-free survival (HR, 0.66; 95% CI, 0.53 to 0.81; *p* < 0.001) [5]. Because of the results of this trial, PC also became a standard of care and was widely adopted in Europe.

These two trials have established either PCR or PC as standard adjuvant therapy for operable stomach cancer. Several other studies have evaluated preoperative chemotherapy with variable results [6,7,8]. A meta-analysis of 12 trials involving 1820 patients that evaluated various preoperative chemotherapy regimens with surgery alone concluded that preoperative chemotherapy was associated with significant improvement in both disease-free survival (odd ratio (OR) 1.85, 95% CI 1.39−2.46) and OS (OR 1.32, 95% CI 1.07−1.64) [9]. Likewise, several randomized trials investigated various postoperative chemotherapy regimens with or without radiation with inconsistent results [10,11,12,13,14]. An individual patient data meta-analysis from 17 trials involving 3838 patients with a median follow-up exceeding seven years demonstrated that adjuvant chemotherapy was associated with a significant benefit in terms of OS (HR, 0.82; 95% CI, 0.76−0.90; *p* < 0.001) and disease-free survival (HR, 0.82; 95% CI, 0.75−0.90; *p* < 0.001) [15]. Another meta-analysis of six randomized controlled trials involving 1171 patients compared adjuvant chemoradiotherapy to chemotherapy [16]. While chemoradiotherapy was associated with a significantly better disease-free survival rate (OR 1.56, 95% CI 1.09−2.24), the survival benefit was not significant (OR 1.32, 95% CI 0.92−1.99). A recent study using U.S. databases suggests that neoadjuvant and perioperative chemotherapy may confer a survival advantage over postoperative treatment [17]; the results have not been confirmed by other studies.

Taken together, it is not known whether the two standard approaches in the management of operable stomach cancer are comparable or if one approach is more effective than the other. There are no randomized trials comparing the two approaches. Moreover, there is limited knowledge about the effectiveness of these two strategies in real-world patients. Despite this, the current trends have led to a greater adoption of PC, especially since the publication of the FLOT (fluorouracil plus leucovorin, oxaliplatin and docetaxel)-4 study [18].

The aim of the current study was to compare the survival of patients with operable stomach cancer who were treated with either PC or PCR as standard adjuvant therapy in Saskatchewan and to determine factors that correlated with survival. We hypothesize that PC improves the outcomes of real-world patients with operable stomach cancer compared to PCR. We also aimed to identify factors that correlated with survival in patients with operable stomach cancer.

## 2. Methods

This was a retrospective population-based cohort study. The study population was comprised of patients with operable stomach cancer diagnosed between January 2005 and December 2015 in the province of Saskatchewan, Canada. International Classification of Disease (ICD) codes relevant for stomach and GE junction cancers were used to identify eligible patients using the Saskatchewan Cancer Registry. Patients with other histological diagnoses, including lymphoma, neuroendocrine tumor, small cell cancer, melanoma and hepatocellular cancer, or with another active secondary malignancy, were excluded. In addition, patients who were treated with preoperative chemoradiotherapy or adjuvant chemotherapy alone were excluded. A pre-specified abstraction sheet was used for data collection. Clinical stage was based on baseline staging imaging scans. All medical records were reviewed and abstracted by a trained research associate. Due to the retrospective nature of the data, only moderate to severe toxicities documented in the health records of patients were recorded.

Based on type of treatment patients were divided into two groups—those who received PC and those who received PCR. PC was mostly comprised of combination chemotherapy that was administered for three cycles preoperatively and three cycles postoperatively. Each 3-week cycle consisted of epirubicin (50 mg/m^2^) by intravenous bolus on day 1, cisplatin (60 mg/m^2^) or oxaliplatin (130 mg/m^2^) intravenously with hydration on day 1, and fluorouracil (200 mg/m^2^) daily for 21 days by continuous intravenous infusion or capecitabine 625 mg/m2/day per oral twice per day (PO BID). PCR was comprised of one cycle of FU (425 mg/m^2^ per day) and leucovorin (20 mg/m^2^ per day) daily for five days then one month later 45 Gy (1.8 Gy per day) of radiation with FU (400 mg/m^2^ per day) and leucovorin (20 mg/m^2^ per day) on days 1 through 4 and on the last three days of RT or capecitabine (825 mg/m^2^ PO BID) continuously during radiation. One month after completion of radiation two more five-day cycles of chemotherapy (FU) 425 mg/m^2^ per day and leucovorin 20 mg/m^2^ per day) were given at monthly intervals.

During the study period both PC and PCR were accepted standard of care treatments. Not all cases were reviewed at a multidisciplinary tumor round. Based on various patients and tumor related factors such as age, underlying cardiovascular disease, or major bleeding, PC or PCR was selected by the treating physicians. All patients who were diagnosed with stomach cancer in Saskatchewan were seen at the two major cancer centers. Patients were followed up at the cancer center after completion of treatment and those who were in remission were subsequently discharged to their primary care physicians. Patients who developed recurrent disease were reassessed for palliative therapy. Survival data was collected from patient’s medical records and the Saskatchewan Cancer Registry.

### Analysis of Primary and Secondary Endpoints

The survivals of the entire cohort and subgroups were estimated by using the Kaplan-Meier method. The survival distribution of different groups was compared by the log rank test. Cox proportional multivariate analysis was performed to determine the prognostic significance of second-line therapy. The HR and its 95% CI were estimated. The following variables were examined with respect to their prognostic significance: age (<65 vs. ≥65); gender; major comorbid illness per the Charlson Comorbidity Index; World Health Organization (WHO) performance status (<1 vs. ≥1); marital status (married or common-law partners vs. single or widowed); children; residence (urban vs. rural); smoking status; cancer center; treatment type (perioperative vs. postoperative chemoradiotherapy); median neutrophil to lymphocyte ratio (NLR); residence; node status (node positive vs. node negative); stage; time period (2005–2010 vs. 2011−2015); and grade (3 or 1 and 2).

All variables with *p* < 0.05 on univariate analysis or which were biologically important were examined in a multivariate model to assess their correlation with survival. The likelihood ratio test and *t*-test were used to determine whether the addition of independent variables of interest significantly added to the prediction of survival in the model. Tests for interaction and confounding factors were assessed for important variables. All *p*-values were two-sided. A *p*-value of <0.05 was considered statistically significant.

## 3. Results

**Figure 1:**
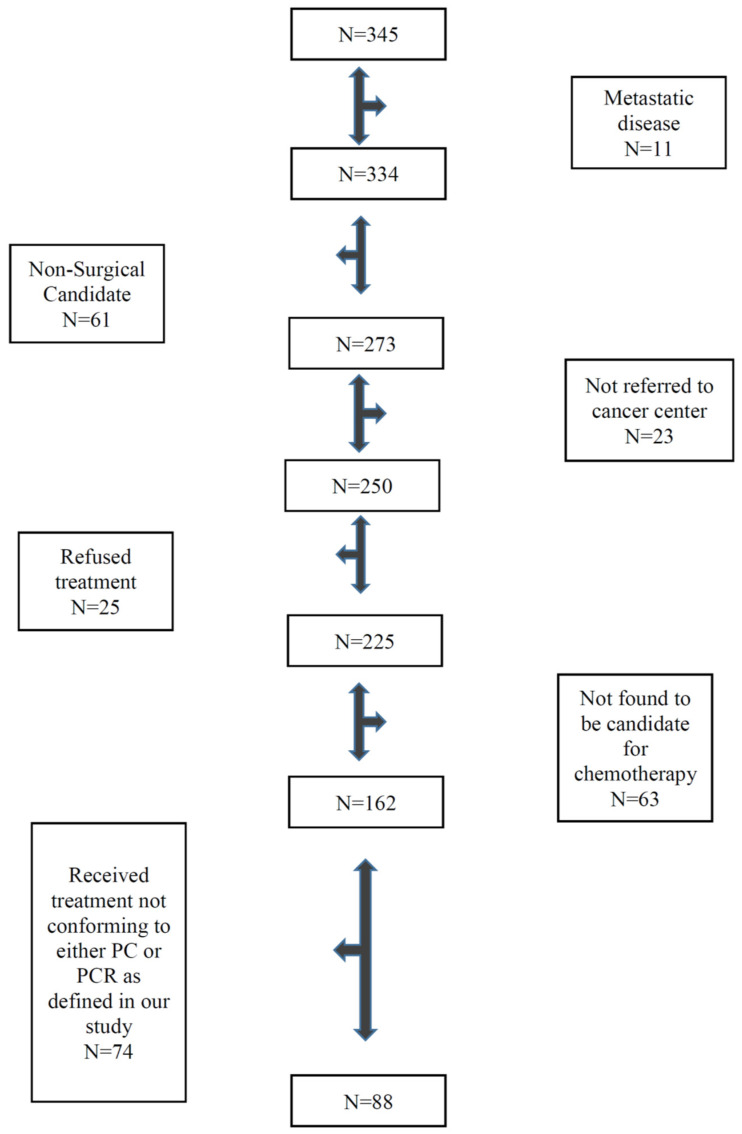
Flow chart showing eligible patients.

Using ICD codes, a total of 345 patients with stomach and GE junction cancers who were diagnosed in Saskatchewan during the study period were identified. Of 345 patients, only 88 patients were found to fulfill eligibility criteria (Figure 1). Most patients were excluded because of metastatic disease, poor performance status, or major active comorbid illnesses, were not candidates for surgery or chemotherapy, or declined standard treatment. In addition, patients with GE junction cancer who were treated with preoperative chemoradiotherapy or those who received postoperative chemotherapy alone were excluded. Of 88 patients, 67 patients received PCR and 21 patients received PC. ECF (epirubicin, cisplatin and infusional 5FU) was the most common regimen and was used in 62% patients followed by EOF (epirubicin, oxaliplatin and infusional 5FU). Only one patient received the FLOT (5FU, oxaliplatin and docetaxel) regimen. Patients’ baseline characteristics are described in Table 1. Despite small numbers, the two groups were relatively well-balanced. The median age of the entire cohort was 63 years. The majority of the patients were men. Most patients had an WHO performance status of 0 or 1, 97% for the PCR group and 100% for the PC group. Most patients had clinical stage I or II cancer, 92% in the PCR group and 85% in the PC group. Post-surgery with or without preoperative chemotherapy pathological stage III was 52% for the entire cohort, 55% in the PCR group vs. 43% in the PC group. About two-thirds of patients had grade 3 tumors.

**Figure 2:**
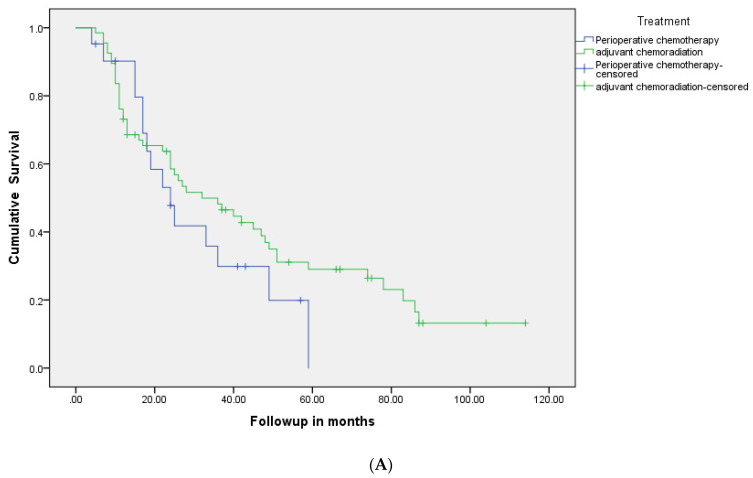

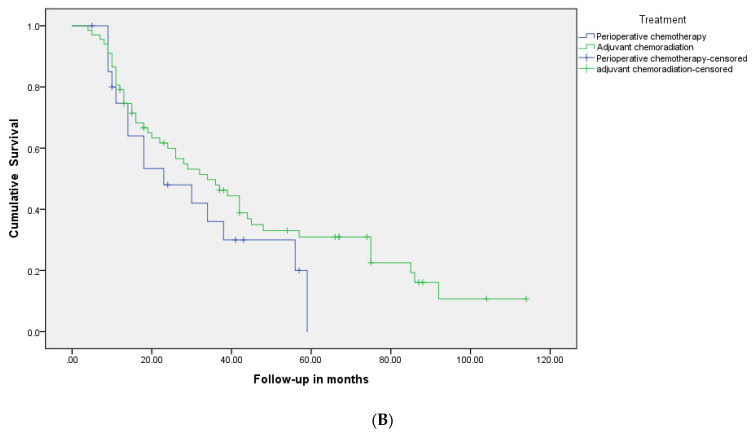
(**A**): Kaplan-Meier relapse-free survival curves of two groups: patients who were treated with perioperative chemotherapy compared to those who received adjuvant chemoradiotherapy. (**B**): Kaplan-Meier overall survival curves of two groups: patients who were treated with perioperative chemotherapy compared to those who received adjuvant chemoradiotherapy.

The OS for the entire group was 34 months (95% CI 23–45), 38 months (24.6–51.3) in the PCR group vs. 30 months (14.3–45.7) in the PC group (*p* = 0.29) (Figure 2A, Table 2). The median relapse-free survival was 34 months for the PCR group and 23 months for the PC group (*p* = 0.20) (Figure 2B). At 5 years, 31% of patients were relapse-free (33% in the PCR group vs. 29% in the PC group) and 34% of patients were alive (39% in the PCR group vs. 29% in the PC group).

On multivariate analysis, T ≥ 3 tumor HR, 3.45 (95% CI: 1.39–8.56), NLR > 2.8, HR, 1.85 (1.05–3.25), and positive resection margin, HR, 1.89 (1.06–3.37) were independently correlated with inferior survival (Table 3). PC did not correlate with better survival, HR, 1.39 (0.71–2.68). Tests for interactions between treatment type and other variables were not significant.

Treatment-related toxicity is described in Table 4. Overall, 34% of patients required hospital admission, 29% in the PCR group vs. 52% in the PC group. The difference did not reach statistical significance. GI toxicity was substantial in both groups. Patients in the PCR group had significantly higher rates of moderate to severe diarrhea (40% in the PCR group vs. 5% in the PC group). The postoperative complication rates were 57% in both groups.

## 4. Discussion

It has been clear for some time that surgery alone is inadequate treatment for stomach cancer. The initial efforts to improve the outcome of surgery alone centered on the INT-0116 trial with adjuvant chemotherapy and radiation. This trial was widely adopted in North America, including Saskatchewan, and at the start of the study period, it was the most common approach for surgically resectable stomach cancers. As the results of the MAGIC trial were reported and later supported by other PC trials, this approach was more widely adopted and gained acceptance in the surgical community [18]. An analysis of the U.S. National Cancer database showed an increase in the use of PC from 7.5% to 46% between 2006 and 2013 [19]. Given that this shift was happening through the later part of our study, it is not surprising that 76% of the study population received PCR as per the INT-0116 protocol.

We noted a high discordance between clinical and pathological staging. Only 7% of the patients were classified as having stage III disease based on preoperative imaging analysis, but this number rose to 52% upon review of surgical pathology. In the early part of the study, CT scans were the main imaging modality for most patients. Later, CT-PET scans were routinely used for staging purposes. Even though CT-PET scans are more effective than CT scans for stomach cancer staging, their sensitivity is limited for nodal staging [20].

Although there is limited data on the comparison between the PC approach and adjuvant chemoradiotherapy, one analysis reported by Jabo et al. that involved 2146 patients showed improved outcomes with PCR over PC [21]. Our results also favor PCR over PC, but these results are statistically insignificant. Our analysis is limited by the low numbers of patients, especially for the PC group. A much larger analysis of the U.S. National Cancer database of over 9000 patients showed no difference between PC and PCR [19]. There was no difference in survival between PC and PCR. Surprisingly, over 40% of patients only had surgery and did not receive additional therapy. This number stayed fairly constant between 2006 and 2013. This study also highlights the need for multidisciplinary discussion in the management of stomach cancer for optimal treatment.

PC has also evolved and improved over time and better results are seen with the FLOT, the newer chemotherapy regimen [18]. In our study, the majority of patients (66%) received the ECF (epirubicin, a platinum compound and 5FU) chemotherapy regimen. Only one patient received FLOT. All of the patients who were designated to receive PC went on to receive all planned preoperative chemotherapy cycles. Even in the postoperative setting, 66% of patients were able to receive the planned chemotherapy. This number is close to the original MAGIC trial where 58% of the subjects assigned to postoperative chemotherapy actually received it. This may be in part due to vigorous patient selection, with 98% of the patients being ECOG 0 or 1. This is something not commonly seen outside the context of clinical trials and may be partly explained by the fact that all patients who were not candidates for chemotherapy were excluded.

The median survival in the INT-0116 trial was 36 months for the PCR group. This cannot be directly compared to the MAGIC trial, as they had different entry points into the trial. This number is very similar to the 38 months (24.6–51.4) seen in our trial. The median survival in the ECF arm in the more recent FLOT study was 35 months. This is better than the OS of 30 months (14.3–45.7) in the PC group. Patients treated with a docetaxel-based FLOT regimen had a median OS of 50 months that has shifted the pendulum in favor of PC in the last few years. Of note, we did not collect treatment information for recurrent disease that could potentially affect survival.

Multivariate analysis also suggested worse outcome with T ≥ 3 and margin positive disease but not with lymph node status. All three of these outcomes have been previously correlated with poorer survival [22,23]. It is likely that the lymph node status was affected by the high rate (>80%) of node positive disease in the entire group, as it impacted the statistical significance of this variable.

Toxicities were similar in both groups. However, patients in the PC group numerically had a high rate of hospital admission. The main toxicities were hematological and gastrointestinal. These two side effects feature prominently in the original INT-0116 trial, as well as the MAGIC trial. Significant postoperative toxicities (57%) in both groups were seen in our study, but PC did not add to additional toxicity at the time of surgery. This was also appreciated in the MAGIC trial where the toxicity in the preoperative chemotherapy group was similar to the surgery alone group (45% in both groups) [5].

Of significant importance, a high baseline NLR was also associated with a poor outcome in the multivariate analysis. This has previously been described as an independent variable in multiple cancers, including gastric cancer as described by Zhang et al. [24]. In this study three-fourths of the patients underwent surgical resection, and a low NLR was associated with an OS benefit (36.0 months vs. 20.5 months, *p* < 0.001). The NLR can be used as an additional metric in risk stratification of newly diagnosed patients with gastric cancer. This may be particularly true for patients who are borderline candidates for surgical resection.

Both treatments are thus feasible and part of international guidelines, although the recent trend is moving towards PC despite there being no randomized trials clearly outlining the superiority of this approach [25,26]. PC does allow systemic disease to be addressed earlier and spares the morbidity of gastrectomy in patients who were destined to do poorly. Given the impressive survival data with FLOT regimen it has established itself as the new standard of care for patients who are fit to receive combination therapy. FLOT based-perioperative therapy has also been adopted as the preferred approach in Saskatchewan for patients with early stomach cancer who are candidates for combination chemotherapy. The role of radiation therapy with FLOT regimen is currently not known and will be determined by future trials. Current clinical trials in early stage stomach cancer are evaluating the efficacy of immunotherapy in combination with chemotherapy and combination of immunotherapy and chemotherapy may become a new standard of care [27].

In conclusion, this well-designed population-based cohort study using individual patient data suggests that both PC and PCR remain feasible approaches for patients in the real-world setting.

## Figures and Tables

**Table 1:** Characteristics of patients who received adjuvant chemoradiotherapy versus perioperative chemotherapy.

Variables	All Patients N = 88 (%)	Adjuvant Chemoradiotherapy Group N = 67 (%)	Peri-Operative Chemotherapy Group N = 21 (%)	*p* Value
Median Age	63 (IQR: 56–71)	63 (IQR: 56–73)	61 (IQR: 52–70)	0.41
Men	61 (69)	48 (72)	13 (62)	0.42
Comorbid illness	51 (58)	40 (60)	11 (52)	0.61
WHO Performance Status				
0	27 (31)	20 (30)	7 (33)	0.79
1	59 (67)	45 (67)	14 (67)	1.0
2	2 (2)	2 (3)	0	1.0
Current Smoker	18 (21)	13 (19)	5 (24)	0.75
Past Smoker	42 (48)	34 (51)	8 (38)	0.33
Secondary Cancer	12 (14)	10 (15)	2 (10)	0.72
Clinical Stage				
I	33 (38)	26 (38)	7 (33)	0.80
II	47 (53)	36 (54)	11 (52)	1.0
III	6 (7)	3 (5)	3 (15)	0.14
Not known	2 (2)	2 (3)	0	
Site				
Gastroesophageal (GE) Junction/cardia	41 (47)	33 (49)	8 (38)	0.45
Body	8 (9)	4 (6)	4 (19)	0.08
Antrum/Pylorus	39 (44)	30 (45)	9 (43)	1.0
Clinical node positive	36 (41)	25 (37)	11 (52)	0.20
Pathological node positive	73 (83)	58 (87)	15 (71)	0.18
Pathological Stage				
0	1 (1)	0	1 (5)	0.23
I	5 (6)	2 (3)	3 (14)	0.08
II	36 (41)	28 (42)	8 (38)	0.80
III	46 (52)	37 (55)	9 (43)	0.45
Positive margin	21 (24)	17 (25)	4 (19)	0.77
Grade ≥ 3	56 (64)	42 (63)	14 (67)	0.80
Mean white blood cell (WBC)	7.7 ± 2.5	7.7 ± 2.7	7.9 ± 2.1	0.68
Mean Hemoglobin	123 ± 19	123 ± 17	125 ± 26	0.52
Mean Platelets	300 ± 102	310 ± 102	271 ± 97	0.12
Mean Creatinine	75 ± 21	76 ± 23	75 ± 17	0.91
Mean Albumin	35 ± 5	35 ± 5	35 ± 5	0.81
Mean Bilirubin	9.1 ± 5.6	9.4 ± 5.9	8.5 ± 4.5	0.54
Mean Alkaline Phosphatase	84 ± 36	81 ± 23	93 ± 65	0.22
Mean Alanine aminotransferase (ALT)	23 ± 35	23 ± 39	23 ± 14	0.98
Mean carcinoembryonic antigen (CEA)	1.8 ± 1.1	1.8 ± 1.1	1.6 ± 1.2	0.63

**Table 2:** Outcome of patients with early stage stomach and GE junction cancers who received adjuvant chemoradiotherapy versus perioperative chemotherapy.

Variables	All Patients N = 88 (%)	Adjuvant Chemoradiotherapy Group N = 67 (%)	Peri-Operative Chemotherapy Group N = 21 (%)	*p* Value
Median RFS months (95% CI)	32 (21.3–42.7)	34 (20.7–47.3)	23 (6.7–39.3)	NS
Median OS months (95% CI)	34 (23–45)	38 (24.6–51.4)	30 (14.3–45.7)	NS
Three year-RFS	49%	55%	43%	NS
Five year-RFS	31%	33%	29%	NS
Three year-OS	52%	55%	48%	NS
Five year-OS	34%	39%	29%	NS

CI: confidence interval; NS: not significance; OS: overall survival; RFS: Recurrence-free survival.

**Table 3:** Univariate and Multivariate Cox Proportional analyses for relationship between various clinical and pathological variables and mortality. A value greater than 1 suggests an increased risk whereas value lower than 1 suggests decreased risk.

Variables	HR (95% CI)			
Univariate			Multivariate	*p*
Age ≥ 70 years	1.01 (0.58–1.73)	0.97	1.10 (0.64–1.88)	0.75
Men	1.11 (0.62–1.97)	0.72	------	
Comorbid illness	1.16 (0.70–1.92)	0.56	1.10 (0.63–1.78)	0.83
WHO performance status > 0	1.45 (0.83–2.53)	0.19	1.48 (0.80–2.71)	0.21
Secondary cancer	0.79 (0.40–1.57)	0.51	------	
Current smoking	1.15 (0.62–2.12)	0.66	------	
Children	0.71 (0.39–1.27)	0.25	------	
Married	0.63 (0.32–1.25)	0.18	------	
Urban City	0.97 (0.58–1.62)	0.92	------	
Neutrophil to lymphocyte ratio (NLR) > 2.80	1.66 (1.01–2.75)	0.048	1.85 (1.05–3.25)	0.034
Node positive	1.47 (0.74–2.89)	0.27	1.31 (0.63–2.71)	0.48
T ≥ 3	3.57 (1.52–8.40)	0.003	3.45 (1.39–8.56)	0.008
Positive margin	2.24 (1.32–3.80)	0.003	1.89 (1.06–3.37)	0.03
Grade 3	1.10 (0.64–1.89)	0.72	------	
Perioperative therapy	1.38 (0.76–2.50)	0.29	1.39 (0.71–2.68)	0.33

Tests for interaction between type of treatment and other variables were not significant and are not showing in the table.

**Table 4:** Treatment toxicities (≥grade 2) in patients with early stage stomach and GE junction cancers who received adjuvant chemoradiotherapy versus perioperative chemotherapy.

Variables	All Patients N = 88 (%)	Adjuvant Chemoradiotherapy Group N = 67 (%)	Peri-Operative Chemotherapy Group N = 21 (%)	*p* Value
Hospital Admission	30 (34)	19 (29)	11 (52)	0.06
Febrile Neutropenia	4 (5)	4 (6)	0	0.56
Neutropenia	11 (13)	9 (13)	2 (10)	1.0
Other infection	6 (7)	6 (9)	0	0.32
Thrombocytopenia	1 (1)	1 (1)	1 (5)	0.42
Anemia	4 (5)	3 (5)	1 (5)	1.0
Nausea	33 (37)	26 (39)	7 (33)	0.79
Vomiting	22 (25)	18 (27)	4 (20)	0.57
Diarrhea	28 (32)	27 (40)	1 (5)	0.002
Neuropathy	5 (6)	3 (5)	2 (10)	0.58
Thromboembolism	3 (3)	2 (3)	1 (5)	0.56
Mucositis	31 (35)	27 (40)	4 (19)	0.11
Fatigue	17 (19)	15 (22)	2 (10)	0.34
Rash	6 (7)	6 (9)	0	0.32
Others	37 (42)	29 (43)	8 (38)	0.80
Post-operative complications	50 (57)	38 (57)	12 (57)	1.0
Wound infection	7 (8)	6 (9)	1 (5)	1.0
Other infection	14 (16)	8 (12)	6 (29)	0.08
Leakage	14 (16)	9 (13)	5 (24)	0.30
Thromboembolism	1 (1)	0	1 (5)	0.23
Cardiovascular events	6 (7)	4 (6)	2 (10)	0.62

## Data Availability

Due to privacy and confidentially data is not available. Part of the data was presented at the European Society of Medical Oncology (ESMO) Annual virtual meeting in September, 2020.
